# Preanalytical Variables in the Analysis of Mitochondrial DNA in Whole Blood and Plasma from Pancreatic Cancer Patients

**DOI:** 10.3390/diagnostics12081905

**Published:** 2022-08-06

**Authors:** Hannah Randeu, Abel J. Bronkhorst, Zsuzsanna Mayer, Angela Oberhofer, Eleni Polatoglou, Volker Heinemann, Michael Haas, Stefan Boeck, Stefan Holdenrieder

**Affiliations:** 1 Munich Biomarker Research Center, Institute of Laboratory Medicine, German Heart Centre, Technical University Munich, D-80636 Munich, Germany; 2 Department of Hematology/Oncology, LMU Klinikum, University of Munich, Comprehensive Cancer Center Munich, D-81377 Munich, Germany

**Keywords:** mitochondrial DNA, preanalytics, cancer, pancreatic cancer, liquid biopsy

## Abstract

Given the crucial role of mitochondria as the main cellular energy provider and its contribution towards tumor growth, chemoresistance, and cancer cell plasticity, mitochondrial DNA (mtDNA) could serve as a relevant biomarker. Thus, the profiling of mtDNA mutations and copy number variations is receiving increasing attention for its possible role in the early diagnosis and monitoring therapies of human cancers. This applies particularly to highly aggressive pancreatic cancer, which is often diagnosed late and is associated with poor prognosis. As current diagnostic procedures are based on imaging, tissue histology, and protein biomarkers with rather low specificity, tumor-derived mtDNA mutations detected from whole blood represents a potential significant leap forward towards early cancer diagnosis. However, for future routine use in clinical settings it is essential that preanalytics related to the characterization of mtDNA in whole blood are thoroughly standardized, controlled, and subject to proper quality assurance, yet this is largely lacking. Therefore, in this study we carried out a comprehensive preanalytical workup comparing different mtDNA extraction methods and testing important preanalytical steps, such as the use of different blood collection tubes, different storage temperatures, length of storage time, and yields in plasma vs. whole blood. To identify analytical and preanalytical differences, all variables were tested in both healthy subjects and pancreatic carcinoma patients. Our results demonstrated a significant difference between cancer patients and healthy subjects for some preanalytical workflows, while other workflows failed to yield statistically significant differences. This underscores the importance of controlling and standardizing preanalytical procedures in the development of clinical assays based on the measurement of mtDNA.

## 1. Introduction

Pancreatic cancer is an extremely malignant and aggressive disease with a limited prognosis and rising incidence, being responsible for an estimated 48,220 deaths in the United States in 2021 [1]. To-date the only curative therapy for pancreatic ductal adenocarcinomas (PDACs) is surgical resection; however, only 15–20% of patients with localized cancer can be cured, while the remaining ~80% have a poor prognosis due to locally advanced or metastasized disease at the time of diagnosis [2,3]. This underscores the importance of early diagnosis and timely treatment. The current diagnostic procedures are based on imaging and tissue histology, as well as on blood-based protein biomarkers such as CEA and CA 19-9 [4]. Although CA 19-9 is a widely used biomarker for the diagnosis of pancreatic cancer, its specificity is rather low. Up to now, blood-based nucleic acid markers have limited use in clinical settings even though they may have a major impact on early detection, monitoring, and treatment by virtue of their disease-specificity, the minimal invasiveness of venipuncture, and the possibility of longitudinal sampling [5,6,7,8]. In recent years, liquid biopsy (also known as liquid profiling), which refers to the analysis of various biomarkers present in human bodily fluids collected with minimally-invasive procedures emerged as a promising diagnostic, prognostic, and theranostic test [2,9,10]. The most commonly characterized liquid biopsy markers in the field of oncology include total cell-free DNA (cfDNA) [5,6,7], circulating tumor DNA (ctDNA) [5,6,7], microRNAs [11], circulating tumor cells (CTCs) [12,13,14,15], and nucleic acid markers associated with extracellular vesicles [16,17]. To-date, the characterization of cell-free nuclear DNA (cf-nDNA) has attracted most attention and has led to the development of several FDA-approved assays, which are currently used in routine clinical practice [18]. While there are currently no FDA-approved assays based on the detection of mitochondrial DNA (mtDNA), a growing body of evidence indicates the potential clinical utility of mtDNA [19,20,21,22,23,24]. In body fluids, mtDNA can be isolated from circulating cells, may be associated with membrane fragments, exist as free-floating fragments, be present in intact mitochondria [25], or encapsulated in microvesicles [26].

Human mitochondria are intracellular organelles composed of eukaryotic, archaeaic, bacterial, and phagic components and play a major role in the production of cellular energy in the form of ATP through the process of oxidative phosphorylation. While the main part of our genetic information is localized in the nucleus, mitochondria contain their own genome within their matrix [22]. MtDNA is a double stranded circular molecule, consisting of 16.569 base pairs, coding for 37 genes that are responsible for proteins of the respiratory chain, tRNAs and rRNAs [27]. Each mitochondrion contains between 2 and 10 copies of their own mtDNA, while the number of mitochondria per cell differs among tissue types, usually reflecting the particular energy requirements of the tissue. This means that many more copies of specific mtDNA are present in a cell as compared to copies of nuclear DNA (nDNA). If released into the blood, changes in mtDNA may therefore be detected more easily and overcome difficulties in finding the rare changes in nDNA deriving from specific cells.

MtDNA content and alterations have been shown to be associated with a wide range of diseases, including cancer development and progression and cardiovascular diseases that are associated with ischemic or hypoxic conditions [21,28,29,30,31,32,33,34,35,36,37]. As mentioned above, mitochondria are vital for cellular energy production. Tumor tissue has an altered energy uptake thus leading to the notion that any pathogenic mutations in mtDNA or copy number variations and further changes in energy production could act as a potential biomarker for several cancer types [28]. This is a consequence of changes in cellular metabolism, the inhibition of the respiratory chain, and the potential increase of reactive oxygen species (ROS) that could initiate tumor growth [5], chemoresistance, and supporting cancer cell plasticity [3,38]. The mtDNA copy number has been reported to vary between cancer and normal tissue cells. It was demonstrated that the mtDNA copy number (mtDNAcn) in tissue from resectable PDACs is lower compared to adjacent normal pancreatic tissue. MtDNAcn and a higher risk of malignancy showed an inverse correlation [38]. Pancreatic cancer originates due to the accumulation of heterogenic, genetic, and epigenetic alterations, but there is also evidence that mutations in mtDNA could be associated with malignant transformation [27,39,40,41,42,43]. Due to a limited number of studies, however, results show different associations between mtDNA copy number and carcinogenesis, as well as no concrete proof that mtDNAcn decrease has an impact on overall survival in resectable PDACs [3].

In light of ongoing studies, mtDNA used in liquid biopsy could potentially serve as a new biomarker for pancreatic cancer patients. As mentioned above, patients are mainly diagnosed in advanced disease states. Through improved diagnosis, prognosis, and therapy monitoring (e.g., in ongoing disease, therapy could be adjusted based on variations in mtDNA parameters enabling personalized treatment), the characterization of mtDNAcn or other potential alterations in mtDNA could, in the future, improve the overall management of pancreatic carcinoma patients.

However, in the many years that it has taken to develop the above-mentioned FDA-approved assays, it has become clear that the development of clinically meaningful liquid biopsy assays, as well as assays that exhibit the necessary sensitivity and specificity required for implementation as a routine diagnostic test, necessitates the rigorous optimization and standardization of preanalytical procedures. It is now well understood that many factors in the long chain of preanalytical steps between sample collection and analysis can greatly impact measurements. Therefore, in order to ensure that laboratory results give the most accurate portrayal of the conditions in the vascular system of the patients [10,44,45], it is essential that preanalytics are rigorously standardized, controlled, and subject to proper quality assurance.

Much research has been done on the preanalytical standardization of cfDNA measurements [46,47,48,49,50], but there is currently a dearth of knowledge on many of the preanalytical variables that impact mtDNA measurements. While the knowledge on cf-nDNA preanalytics is to some extent translatable to mtDNA analysis, more studies specifically on mtDNA are very much needed as these molecules are not protected by histone proteins, are more vulnerable to digestion, and exhibit very different dynamics in the extracellular space. In addition, mtDNA can be assessed either in whole blood or in blood plasma, which poses different challenges in preanalytical sample handling.

Therefore, in this work we compared the yield between two mtDNA extraction methods and assessed several important preanalytical steps such as the use of two different blood collection tubes, different storage temperatures, and yields in plasma or whole blood. To identify analytical and preanalytical differences, these variables were all tested in the blood of healthy subjects and pancreatic carcinoma patients. Our study setting is a substantial precondition for a standardized and quality-controlled use of methods in clinical biomarker studies.

## 2. Materials and Methods

### 2.1. Subjects and Ethics Approval Statement

The blood of 10 healthy individuals and 10 patients who were treated for pancreatic cancer at the Department of Internal Medicine III and Comprehensive Cancer Center at the University Hospital Munich-Grosshadern was obtained after written consent was given by the blood donors. The study was approved by the ethics committee of the Ludwig-Maximilians-Universität München (Project Nr: 21-0707). Furthermore, 10 samples of the RASH (*n* = 150) and ACCEPT (*n* = 119) studies (published therapy studies in which patients with metastasized pancreatic cancer were recruited) were used [51,52].

### 2.2. Variables Evaluated in This Study

We evaluated different preanalytical variables as it relates to the sample type, processing procedures, and the extraction of both genomic and mtDNA from human biospecimens. We compared the total yield of mtDNA (i) in whole blood vs. plasma, (ii) when stored at room temperature (RT) vs. frozen at −20 °C for six days prior to processing, (iii) when collected in PAXgene Blood DNA vs. EDTA tubes, (iv) when isolated with two different extraction kits, and (v) in samples collected in 2014–2015 vs. samples collected in 2021. nDNA was measured as a control in selected experiments.

### 2.3. Sample Collection and Processing

A total of 35 mL of venous blood was collected by venipuncture in two K3-EDTA tubes (S-Monovette^®^ KALIUM-EDTA, Sarstedt AG & Co., Nümbrecht, Germany) of 9 mL and two PAXgene™ Blood DNA tubes (PreAnalytiX GmbH, Hombrechtikon, Switzerland) of 8.5 mL. After two hours of storage at room temperature one EDTA and one PAXgene Blood DNA tube each were spun at 1600× *g* for 10 min at room temperature. Plasma was removed in falcon tubes, without disturbing the buffy coat, and respun at 2800× *g* for 15 min to remove residual cellular debris. Whole blood and obtained plasma of both tubes was sub-aliquoted into 1.5 mL microcentrifuge tubes (DNA LoBind^®^ Tubes, Eppendorf, Hamburg Germany) and stored at −20 °C.

### 2.4. Isolation of DNA

For all samples in this study, mtDNA was isolated with a magnetic bead-based, partially automated extraction method using the Wizard^®^ Plus SV Minipreps DNA purification system followed by automated extraction with the Promega Maxwell instrument. The samples were prepared with a red blood cell lysis solution, and pelleted white cells were treated with a cell resuspension solution, cell lysis solution and a neutralization solution. After automated extraction, the DNA samples were eluted in 60 μL elution buffer. This method was compared with a manual column-based plasmid extraction kit (QIAprep^®^ Miniprep Kit). The manual kit is based on three main steps: (i) cell lysis and neutralization, (ii) plasmid DNA binding on the silica membrane, and (iii) finishing with washing steps and elution in 50 μL of elution buffer. Of the sample, 1 mL was processed for the plasmid kit in duplicate, and 0.5 mL was used for the automated kit.

### 2.5. Quantification of Mitochondrial and Nuclear DNA

nDNA and mtDNA was amplified using a 96 well-plate on the LightCycler 480^®^ System (Roche Diagnostics International AG, Rotkreuz, Switzerland). The reaction mixture consisted of 1 μL sample, 800 μL master mix, 680 μL nuclease free water, and 20 μL of forward and reverse primer (20 μM) each. The primers used for mtDNA were MT-TL1: F1, 5′CACCCAAgAACAgggTTTgT-3′; R1, 5′-TggCCATgggTATgTTgTTA-3′. The primers used for nuclear DNA were β-globin: F1, 5′-gAAgAgCCAAggACAggTAC-3′; R1, 5′-CAACTTCATCCACgTTCACC-3′. These primers were synthesized by TIB Molbiol (TIP Molbiol GmbH, Berlin, Germany). PCR conditions were set to 95 °C for 60 s, followed by 40 cycles of 15 s denaturation at 95 °C, 30 s annealing at 58 °C, followed by 30 s extension at 60 °C. The absolute concentration of the target gene was calculated using a standard curve, which was based on a dilution series measured with qPCR. The mentioned standard curve and copy number calculation was performed as follows: PCR was performed using MT-TL1 and β-globin primers, and the DNA cleanup was performed using the DNA CleanUp Monarch DNA Kit. Fragment size distribution was assessed using the Bioanalyzer DNA 1000 kit (Agilent Technologies Inc., Santa Clara, CA, USA), and the DNA enrichment was measured using the Qubit dsDNA HS Assay. The copy number was then calculated using the mtDNA concentrations and the base pairs were determined by the Bioanalyzer (Number of copies = (amount × 6.022 × 10^23^)/(length × 1 × 10^9^ × 660)). Samples were measured in triplicates for both assays, enabling the calculation of the average mtDNA to nDNA ratio.

### 2.6. Statistical Analysis

All statistics and data visualization were performed using GraphPad Prism version 9 for Windows (GraphPad Software, San Diego, CA, USA, www.graphpad.com (accessed on 22 June 2022)) Grubbs’ test was used to identify outliers. Only *p*-values < 0.05 were considered to be statistically significant.

## 3. Results and Discussion

In this study we evaluated different preanalytical variables as it relates to the sample type, processing procedures, and extraction of both nuclear and mtDNA from whole blood and plasma of healthy subjects and a cohort of pancreatic cancer patients.

### 3.1. Whole Blood vs. Plasma

**Figure 1:**
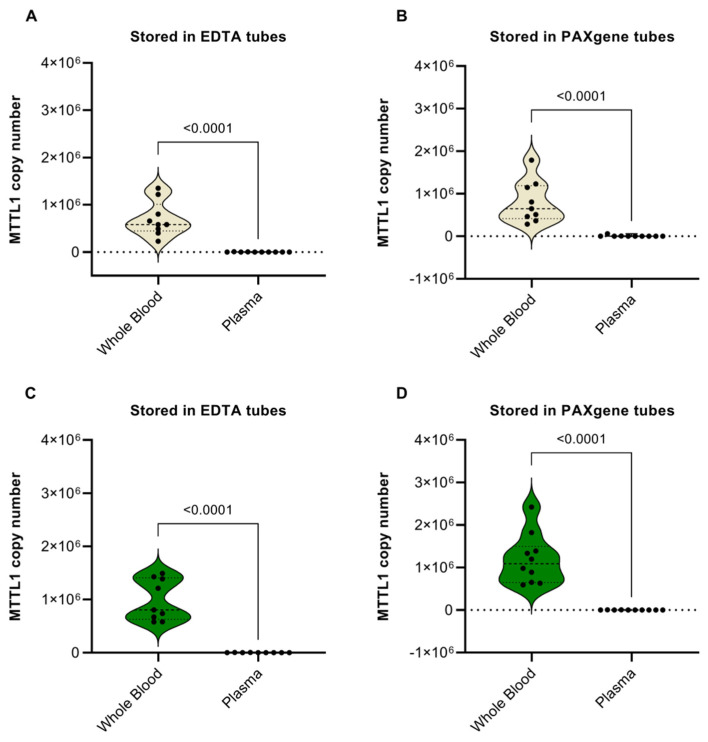
Mitochondrial DNA levels in whole blood vs. plasma. Violin plots showing the comparison of mitochondrial DNA (mtDNA) copy number in whole blood and plasma samples from healthy subjects stored in (**A**) EDTA and (**B**) PAXgene tubes and in pancreatic cancer patients stored in (**C**) EDTA and (**D**) PAXgene tubes. MtDNA was obtained by semi-automated isolation using the Maxwell RSC Instrument and quantified by an MT-TL1 qPCR assay. Ten subjects were recruited in each cohort. Significant outliers were identified and omitted using Grubb’s test (Alpha = 0.05). *p*-values < 0.05 indicate a statistically significant difference.

Significantly higher levels of mtDNA were observed in whole blood as compared with blood plasma for all conditions being stored in EDTA or PAXgene tubes and being tested in healthy subjects (Figure 1A,B) and cancer patients (Figure 1C,D). The reason for this is likely that the main part of the mtDNA originates from white blood cells, which are only present in whole blood but are already removed in plasma.

### 3.2. Sample Storage at Room Temperature vs. −20 °C Prior to Processing

**Figure 2:**
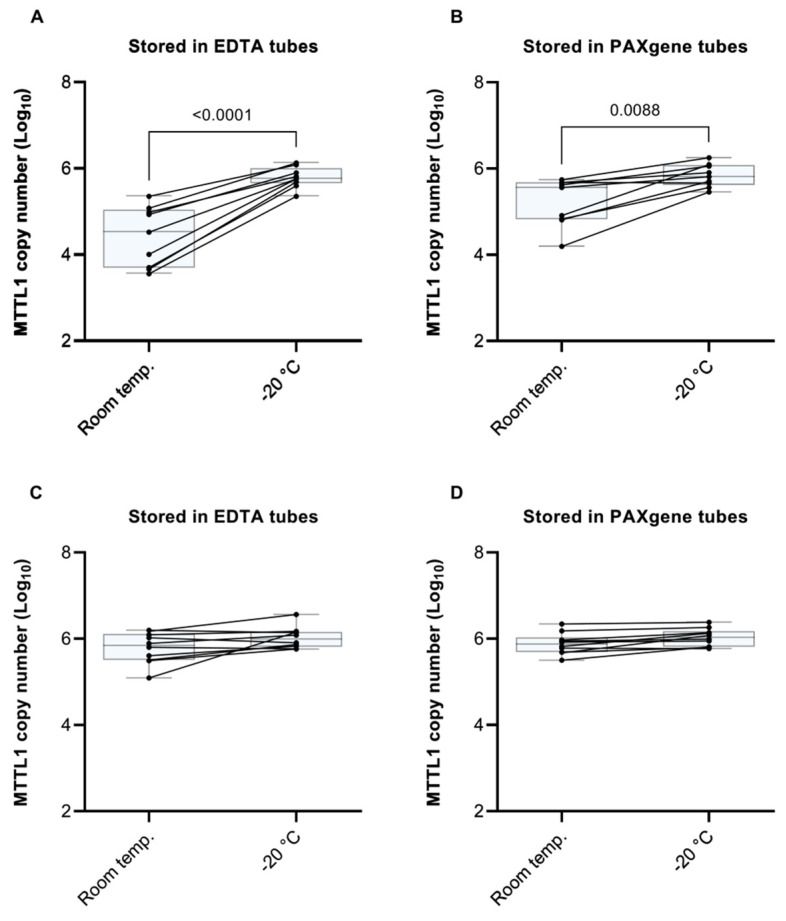
The effect of storage temperature on mitochondrial (mtDNA) levels. Top part (**A**,**B**): Comparison of mtDNA copy number present in whole blood samples of healthy subjects stored at room temperature (RT) vs. −20 °C prior to processing in (**A**) EDTA tubes and (**B**) PAXgene tubes. Bottom part (**C**,**D**): Comparison of mtDNA copy number present in whole blood samples of pancreatic cancer patients stored at room temperature (RT) vs. −20 °C prior to processing in (**C**) EDTA tubes and (**D**) PAXgene tubes. Part 2 (**C**,**D**): MtDNA was obtained by semi-automated isolation using the Maxwell RSC Instrument and quantified by an MTTL1 qPCR assay. Ten subjects were recruited in each cohort. Significant outliers were identified and omitted using Grubb’s test (Alpha = 0.05). The fold-change in the copy number of MTTL1 for each patient is summarized in Supplementary Table S1.

In the case of healthy patients (Figure 2A,B), storage of whole blood samples in both EDTA and PAXgene tubes at −20 ℃ delivered a significantly higher yield of mtDNA vs. storage at RT prior to processing. This difference was more pronounced in EDTA tubes than in PAXgene tubes, which is likely due to the protective effects of the DNA-stabilizing agent present in PAXgene tubes. The observation that storage at −20 °C vs. RT prior to sample processing delivered a higher yield may be explained by the fact that a major part of white blood cells bursts during freezing and thawing procedures. Interestingly, however, in the case of cancer patient samples (Figure 2C,D), sample storage temperature prior to processing had virtually no effect on mtDNA yield. A possible reason for this difference between healthy subjects and cancer patients may be a higher degree of cell lysis in cancer patients due to the presence of different or higher amounts of enzymes, cytokines, lipids or apoptosis-promoting factors, and increased cell turnover owing to chronic infection. The fold-change in mtDNA copy number present in whole blood samples stored at RT vs. −20 °C prior to processing of all experimental subjects is summarized in Supplementary Table S1.

### 3.3. EDTA vs. PAXgene Tubes

**Figure 3:**
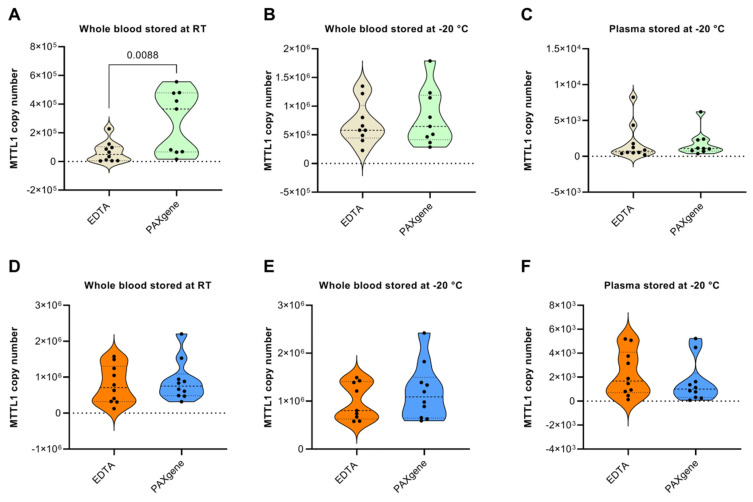
EDTA vs. PAXgene tubes for the storage of mitochondrial DNA (mtDNA). MtDNA copy number in whole blood of healthy subjects stored in EDTA vs. PAXgene tubes at (**A**) RT for 3 h prior to processing and (**B**) −20 °C prior to processing. (**C**) Comparison of mtDNA copy number in plasma samples of healthy subjects stored in EDTA vs. PAXgene tubes at −20 °C prior to processing. MtDNA copy number in whole blood of pancreatic cancer patients stored in EDTA vs. PAXgene tubes at (**D**) RT for 3 h prior to processing and (**E**) −20 °C prior to processing. (**F**) Comparison of mtDNA copy number in plasma samples of healthy subjects stored in EDTA vs. PAXgene tubes at −20 °C prior to processing. MtDNA was obtained by semi-automated isolation using the Maxwell RSC Instrument and quantified by an MTTL1 qPCR assay. Ten subjects were recruited in each cohort. Significant outliers were identified and omitted using Grubb’s test (Alpha = 0.05). *p*-values < 0.05 indicate a statistically significant difference.

In both healthy subjects (Figure 3A–C) and cancer patients (Figure 3D–F), there were no statistically significant differences in the yield of mtDNA when stored in PAXgene vs. EDTA tubes for any of the tested conditions, with the exception of whole blood stored in PAXgene tubes at room temperature prior to processing, which delivered a higher yield of mtDNA compared to EDTA tubes (Figure 3A). A possible explanation for the latter is that the mtDNA concentration at RT is very low because the main part of mtDNA in whole blood originates from white blood cells, and as mentioned earlier, these low amounts could potentially be preserved to a greater extent in PAXgene Blood DNA tubes that contain reagents that stabilize DNA.

### 3.4. QIAprep vs. Maxwell Isolation Kits

**Figure 4:**
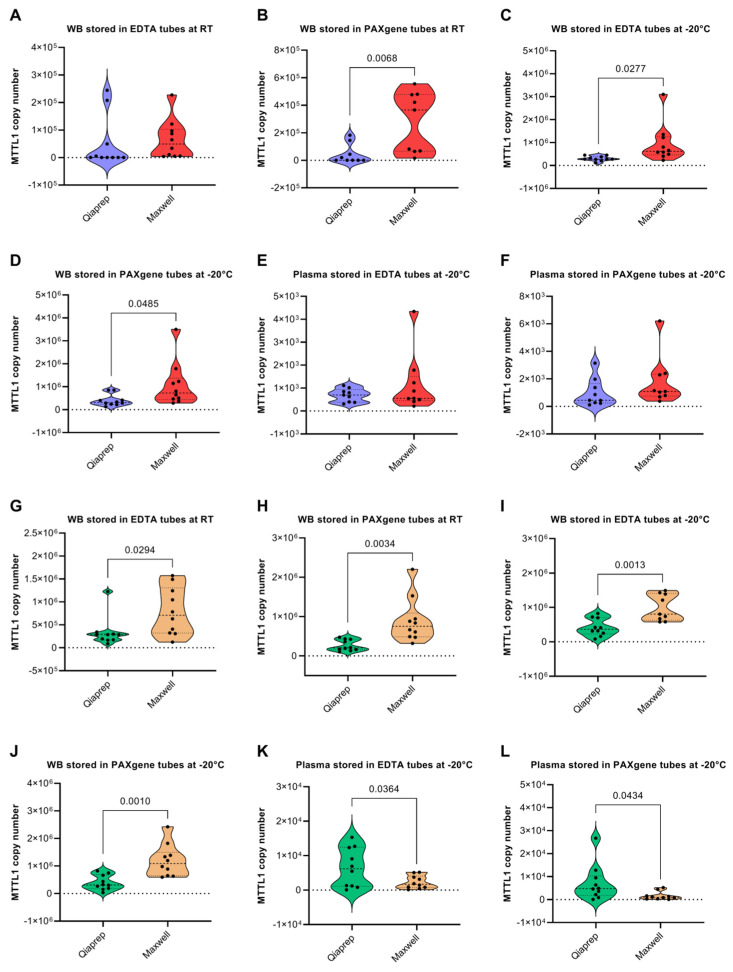
Comparison of methods for the isolation of mtDNA from whole blood and plasma. Comparison of mtDNA copy number present in whole blood samples of healthy subjects isolated with the Maxwell and QIAprep kit stored in (**A**) EDTA tubes at RT for 3 h prior to processing, (**B**) PAXgene tubes at RT for 3 h prior to processing, (**C**) EDTA tubes at −20 °C prior to processing, (**D**) PAXgene tubes at −20 °C prior to processing. Comparison of mtDNA copy number present in plasma samples of healthy subjects isolated with the Maxwell and QIAprep kit stored in (**E**) EDTA tubes at −20 °C prior to processing, (**F**) PAXgene tubes at −20 °C prior to processing. Comparison of mtDNA copy number present in whole blood samples of pancreatic cancer patients isolated with the Maxwell and QIAprep kits stored in (**G**) EDTA tubes at RT for 3 h prior to processing, (**H**) PAXgene tubes at RT for 3 h prior to processing, (**I**) EDTA tubes at −20 °C prior to processing, (**J**) PAXgene tubes at −20 °C prior to processing. Comparison of mtDNA copy number present in plasma samples of pancreatic cancer patients isolated with the Maxwell and QIAprep kit stored in (**K**) EDTA tubes at −20 °C prior to processing, (**L**) PAXgene tubes at −20 °C prior to processing. Ten subjects were recruited in each cohort. Significant outliers were identified and omitted using Grubb’s test (Alpha = 0.05). *p*-values < 0.05 indicate a statistically significant difference. Kit comparisons in terms of the yield of nDNA in whole blood (as measured by a β-globin qPCR assay) is shown in Supplementary Figure S1, while the ratio of MTTL1 to β-globin recovered by the different kits in the different conditions is shown in Supplementary Figure S2.

In healthy patients, the Maxwell kit delivered significantly higher yields of mtDNA than the QIAprep kit when isolated from whole blood in most cases, except no significant difference between the yields of mtDNA was observed between the two kits when samples were stored in EDTA tubes at room temperature (Figure 4A). This may be due to the fact that the QIAprep kit isolates only circular DNA, while the Maxwell kit extracts less specifically and more automated. Also, as mentioned above, PAXgene tubes stabilize genomic DNA better than EDTA tubes. Conversely, there was no significant difference in the yield of mtDNA delivered by the two kits when mtDNA was isolated from plasma samples (Figure 4E,F). Perhaps this lack of difference in mtDNA yield from plasma samples between the two kits is due to the low levels of mtDNA present in plasma samples. In contrast to healthy patients, the QIAprep kit delivered significantly higher yields of mtDNA vs. the Maxwell kit when mtDNA was isolated from plasma samples in cancer patients (Figure 4K,L). The difference between healthy subjects and cancer patients may be due to a higher sensitivity of the QIAprep kit towards lower amounts of mtDNA, and that the mtDNA yield in plasma of cancer patients is possibly higher than in healthy samples (as shown later in Section 3.6. Conversely, in all of the conditions tested the Maxwell kit delivered significantly higher yields of mtDNA than the QIAprep kit when mtDNA was isolated from whole blood in cancer patients (Figure 4G–J).

Nuclear DNA levels, as measured with a β-globin qPCR assay, were low and highly variable in comparison with mtDNA levels. This is expected as these extraction methods are tailored for the isolation of mtDNA and not nDNA. Low nDNA levels are preferred for downstream analyses, such as mtDNA sequence analysis. In both healthy subjects (Supplementary Figure S1A,B) and cancer patients (Supplementary Figure S1E,F) there was no significant difference in the recovery of nDNA between the different kits when isolated from whole blood stored in either EDTA or PAXgene tubes at RT prior to processing. However, in both healthy subjects (Supplementary Figure S1C,D) and cancer patients (Supplementary Figure S1G,H), the Maxwell kit recovered significantly higher levels of nDNA from samples stored in both EDTA and PAXgene tubes at −20 °C prior to processing. Therefore, the Maxwell kit displays superior recovery efficiency for both nDNA and mtDNA.

Concerning the ratio of nuclear to mtDNA recovered by the different kits, the Maxwell kit recovered a higher ratio of mtDNA from whole blood samples of healthy patients stored in both EDTA and PAXgene tubes at RT prior to processing (Supplementary Figure S2A,B). In contrast, there were no significant differences between the two kits for samples stored in either EDTA or PAXgene tubes at −20 °C prior to processing (Supplementary Figure S2C,D). In cancer patients, the Maxwell kit delivered a significantly higher ratio of mtDNA to nDNA than the QIAprep kit only in whole blood samples stored in PAXgene tubes at RT prior to processing (Supplementary Figure S2F). Interestingly, in contrast to healthy subjects, in cancer patients the QIAprep kit recovered a significantly higher mtDNA to nDNA ratio in whole blood samples stored in both EDTA and PAXgene tubes stored at −20 °C prior to processing (Supplementary Figure S2G,H). Taken together, this indicates that the QIAprep delivers a purer mtDNA sample, while the Maxwell kit delivers a higher yield of mtDNA.

### 3.5. Effect of Sample Storage Time on the Yield of mtDNA and nDNA

**Figure 5:**
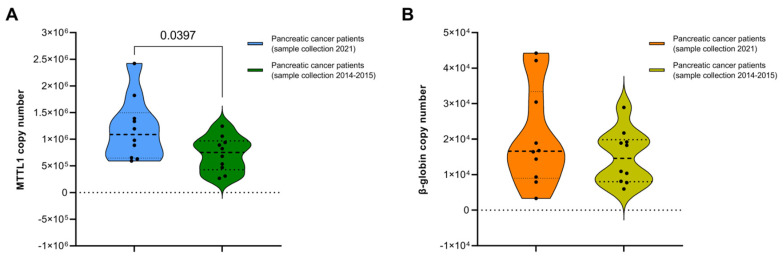
The effect of sample storage time on the yield of mitochondrial DNA and nuclear DNA. Comparison of mtDNA and nDNA present in whole blood samples of pancreatic cancer patients with sample collection in 2021, stored at −20 °C prior to processing, vs. 2014–2015, stored at −80 °C prior to processing. Comparison of (**A**) mtDNA and (**B**) nDNA in whole blood samples, followed by mtDNA isolation with the Maxwell Kit. Ten patients were recruited in each cohort. Outliers were identified and omitted using Grubb’s test (Alpha = 0.05). *p*-values lower than 0.05 indicate a statistically significant difference.

Regarding the mtDNA yield of pancreatic cancer patients with sample collection in 2021 vs. in 2014–2015 (Figure 5A), significantly higher levels of mtDNA was observed in the more recently recruited cohort. In contrast, nDNA showed no significant differences between the two sample sets (Figure 5B). This could be due to differences in stability of nDNA and mtDNA, e.g., a lack of histone packaging of mtDNA and storage at lower (−20 vs. −80 °C prior to processing) temperatures. These results imply that the duration of storage has a significant impact on the mtDNA sample yield. Whether it has an impact on the quality or for instance epigenetic factors is unclear. While studies show that nDNA stored at −80 °C can be used for further molecular experiments for many years [53], it is yet to be investigated how mtDNA yields and quality changes over storage time.

### 3.6. Comparison of Cancer Patients and Healthy Subjects

**Figure 6:**
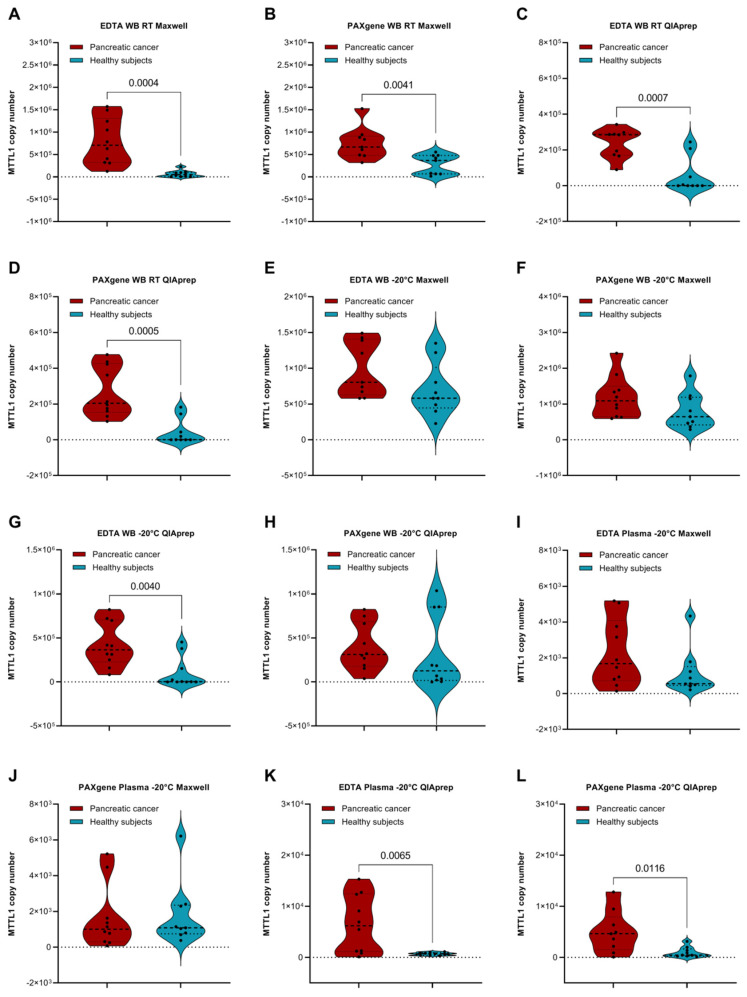
Comparison of mtDNA levels in pancreatic cancer patients vs. healthy subjects under various preanalytical conditions. Comparison of mtDNA copy number present in whole blood samples of healthy subjects vs. cancer patients stored in EDTA vs. PAXgene tubes at RT for 3 h prior to processing, followed by mtDNA isolation with (**A**,**B**) the Maxwell Kit and (**C**,**D**) the QIAprep Kit, as well as with storage of −20 °C prior to processing, followed by mtDNA isolation with (**E**,**F**) the Maxwell Kit, and (**G**,**H**) the QIAprep Kit. Comparison of mtDNA copy number present in plasma samples of healthy subjects vs. cancer patients stored in EDTA vs. PAXgene tubes at −20 °C prior to processing, followed by mtDNA isolation with the Maxwell Kit (**I**,**J**) and (**K**,**L**) the QIAprep Kit. Ten healthy subjects were recruited in each cohort. Outliers were identified and omitted using Grubb’s test (Alpha = 0.05). *p*-values lower than 0.05 indicate a statistically significant difference.

We compared mtDNA levels in healthy subjects and cancer patients under different preanalytical conditions (Figure 6). Statistically significant higher levels were found in whole blood samples of cancer patients vs. healthy subjects when stored at RT, regardless of which tube or extraction method was used (Figure 6A–D). This could be due to a higher amount of extracellular and cell-free mtDNA or a higher cell turnover in blood of cancer patients due to a different composition and presence of enzymes, lipids or apoptosis-promoting factors. In contrast, storage of whole blood samples in either EDTA or PAXgene tubes at −20 °C prior to processing did not yield significantly different mtDNA measurements between cancer patients and healthy subjects (Figure 6E,F,H), except in the case of whole blood stored in EDTA tubes followed by mtDNA extraction using the QIAprep kit (Figure 6G). This may be due to single healthy individuals with high mtDNA concentrations. Concerning plasma samples stored at −20 °C prior to processing, significantly higher mtDNA levels were found in cancer patients vs. healthy subjects in samples stored in either EDTA or PAXgene tubes, followed by isolation using the QIAprep kit (Figure 6K,L). In contrast, there were no statistically significant differences observed between cancer patients and healthy subjects in samples isolated with the Maxwell kit (Figure 6I,J). The fact that only the QIAprep Kit yields more mtDNA can be due to the higher sensitivity of this Kit in Plasma and only slightly raised levels of mtDNA.

As the extraction kits used in this study are tailored for the extraction of mtDNA and delivered very low and inconsistent nDNA yields, we did not compare nDNA measurements between cancer patients and healthy subjects.

## 4. Conclusions

MtDNA mutations and copy number variations have been correlated with various pathological indications in many types of human cancer, making the detection of mtDNA in body fluids an ideal candidate biomarker for the diagnosis and monitoring of disease progression. However, the development of clinically meaningful mtDNA-based liquid biopsy tests necessitates the optimization and standardization of preanalytical procedures.

Here we summarize the observations made in our evaluation of several preanalytical variables: (i) whole blood contains significantly higher levels of mtDNA than plasma, while ratios of mtDNA/nDNA in plasma are generally higher than in WB, which may be due to a smaller amount of nDNA in plasma or the underestimation of nDNA levels due to inefficient extraction of nDNA by kits tailored for mtDNA extraction; (ii) higher yields are generally achieved when samples were stored at −20 °C before processing; (iii) PAXgene tubes may be better suited for extractions at RT vs. EDTA tubes. Apart from this, there were no significant differences observed between EDTA and PAXgene tubes; (iv) extraction with the Maxwell Kit out of WB delivers higher yields of nDNA in general, but also higher mtDNA levels overall, which may be essential for meeting the high input demands of downstream analyses, such as NGS; (v) the QIAprep Kit yields less nuclear DNA and therefore has a higher ratio of mtDNA/nDNA but captured less absolute mtDNA than the Maxwell Kit. Choosing between these two kits depends on the aims of the experiment in question, e.g., the necessity of a high ratio of mt/nDNA and primarily “pure” mtDNA, on the impact of the presence of nDNA on further processing, or on the importance of a high mtDNA yield for downstream experiments; (vi) In view of the fact that stable sample material is indispensable for the reliability of analytical conclusions, it is also necessary to take into account that the storage time of blood between two samples can cause variations in copy number comparisons; and lastly (vii) when comparing mtDNA levels in pancreatic cancer patients vs. healthy subjects under various preanalytical conditions, more mtDNA is yielded when extracting at RT or from plasma. This indicates that there is more extracellular mtDNA in the blood of pancreatic cancer patients. However, it is also clear that this correlation only exists when specific preanalytical procedures are followed. Thus, a conclusion cannot be confidently drawn. This shows that future studies on the standardization of mtDNA extraction and all relevant preanalytical procedures are crucial in order to maximize the reliability of measurements and allow complete access to non-invasively investigate changes in the mitochondrial genome.

## Data Availability

Not applicable.

## Supplementary Materials

The following supporting information can be downloaded at: https://www.mdpi.com/article/10.3390/diagnostics12081905/s1, Figure S1: Comparison of methods for the isolation of nuclear DNA from whole blood and plasma; Figure S2: Comparison of the ratio of mtDNA/nDNA recovered by different isolation methods; Supplementary Table S1: Mitochondrial DNA copy number (CN) in WB stored at different temperatures prior to processing.
