# Supplementary material for: Preanalytical Variables in the Analysis of Mitochondrial DNA in Whole Blood and Plasma from Pancreatic Cancer Patients
Source: Diagnostics (Basel). 2022 Aug 6;12(8):1905. doi: 10.3390/diagnostics12081905 (PMC9406772; doi:10.3390/diagnostics12081905)
Supplement: Supplementary file 1 [file diagnostics-12-01905-s001.zip › Supplementary Figure S1.pdf]

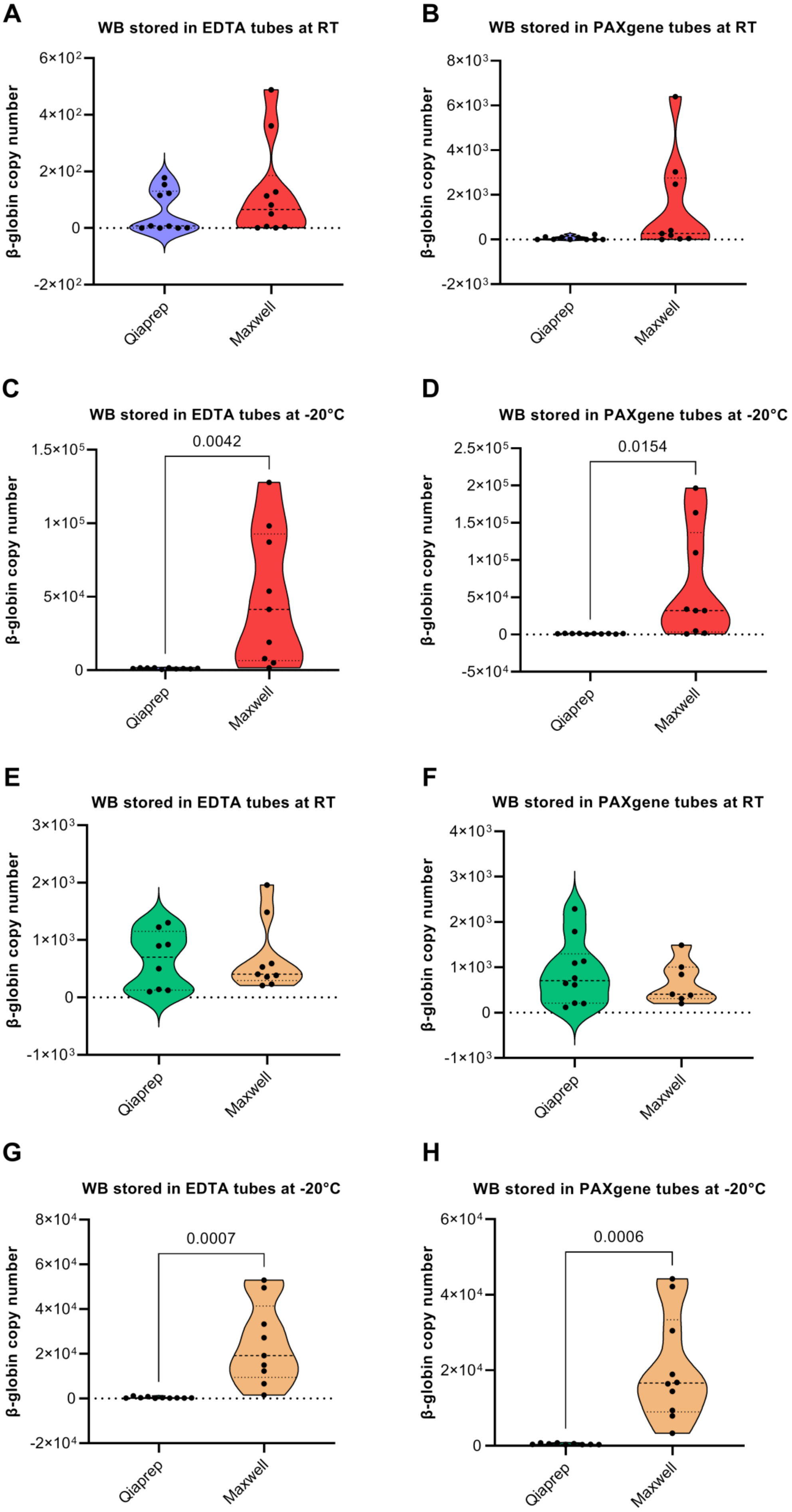

A
WB stored in EDTA tubes at RT
β-globin copy number
6×10²
4×10²
2×10²
0
-2×10²
Qiaprep
Maxwell
B
WB stored in PAXgene tubes at RT
β-globin copy number
8×10³
6×10³
4×10³
2×10³
0
-2×10³
Qiaprep
Maxwell
C
WB stored in EDTA tubes at -20°C
0.0042
β-globin copy number
1.5×10⁵
1×10⁵
5×10⁴
0
Qiaprep
Maxwell
D
WB stored in PAXgene tubes at -20°C
0.0154
β-globin copy number
2.5×10⁵
2×10⁵
1.5×10⁵
1×10⁵
5×10⁴
0
-5×10⁴
Qiaprep
Maxwell
E
WB stored in EDTA tubes at RT
β-globin copy number
3×10³
2×10³
1×10³
0
-1×10³
Qiaprep
Maxwell
F
WB stored in PAXgene tubes at RT
β-globin copy number
4×10³
3×10³
2×10³
1×10³
0
-1×10³
Qiaprep
Maxwell
G
WB stored in EDTA tubes at -20°C
0.0007
β-globin copy number
8×10⁴
6×10⁴
4×10⁴
2×10⁴
0
-2×10⁴
Qiaprep
Maxwell
H
WB stored in PAXgene tubes at -20°C
0.0006
β-globin copy number
6×10⁴
4×10⁴
2×10⁴
0
Qiaprep
Maxwell

Supplementary Figure S1. Comparison of methods for the isolation of nuclear DNA from whole blood and plasma. Comparison of β-Globin copy number present in whole blood samples of healthy subjects isolated with the Maxwell and Qiagen kit stored in (**A**) EDTA tubes at RT for 3h prior to processing, (**B**) PAXgene tubes at RT for 3h prior to processing, (**C**) EDTA tubes at -20 °C prior to processing, (**D**) PAXgene tubes at -20 °C prior to processing. Comparison of mtDNA copy number present in whole blood samples of pancreatic cancer patients isolated with the Maxwell and Qiaprep kits stored in (**E**) EDTA tubes at RT for 3h prior to processing, (**F**) PAXgene tubes at RT for 3h prior to processing, (**G**) EDTA tubes at -20 °C prior to processing, (**H**) PAXgene tubes at -20 °C prior to processing. Ten subjects were recruited in each cohort. Significant outliers were identified and omitted using Grubb's test (Alpha = 0.05). P-values < 0.05 indicate a statistically significant difference. Kit comparisons in terms of the yield of nuclear DNA in whole blood (as measured by a β-globin qPCR assay) is shown in Supplementary Figure S1, while the ratio of MTTL1 to β-globin recovered by the different kits in the different conditions is shown in Supplementary Figure S2.
