# Supplementary material for: Preanalytical Variables in the Analysis of Mitochondrial DNA in Whole Blood and Plasma from Pancreatic Cancer Patients
Source: Diagnostics (Basel). 2022 Aug 6;12(8):1905. doi: 10.3390/diagnostics12081905 (PMC9406772; doi:10.3390/diagnostics12081905)
Supplement: Supplementary file 1 [file diagnostics-12-01905-s001.zip › Supplementary Figure S2.pdf]

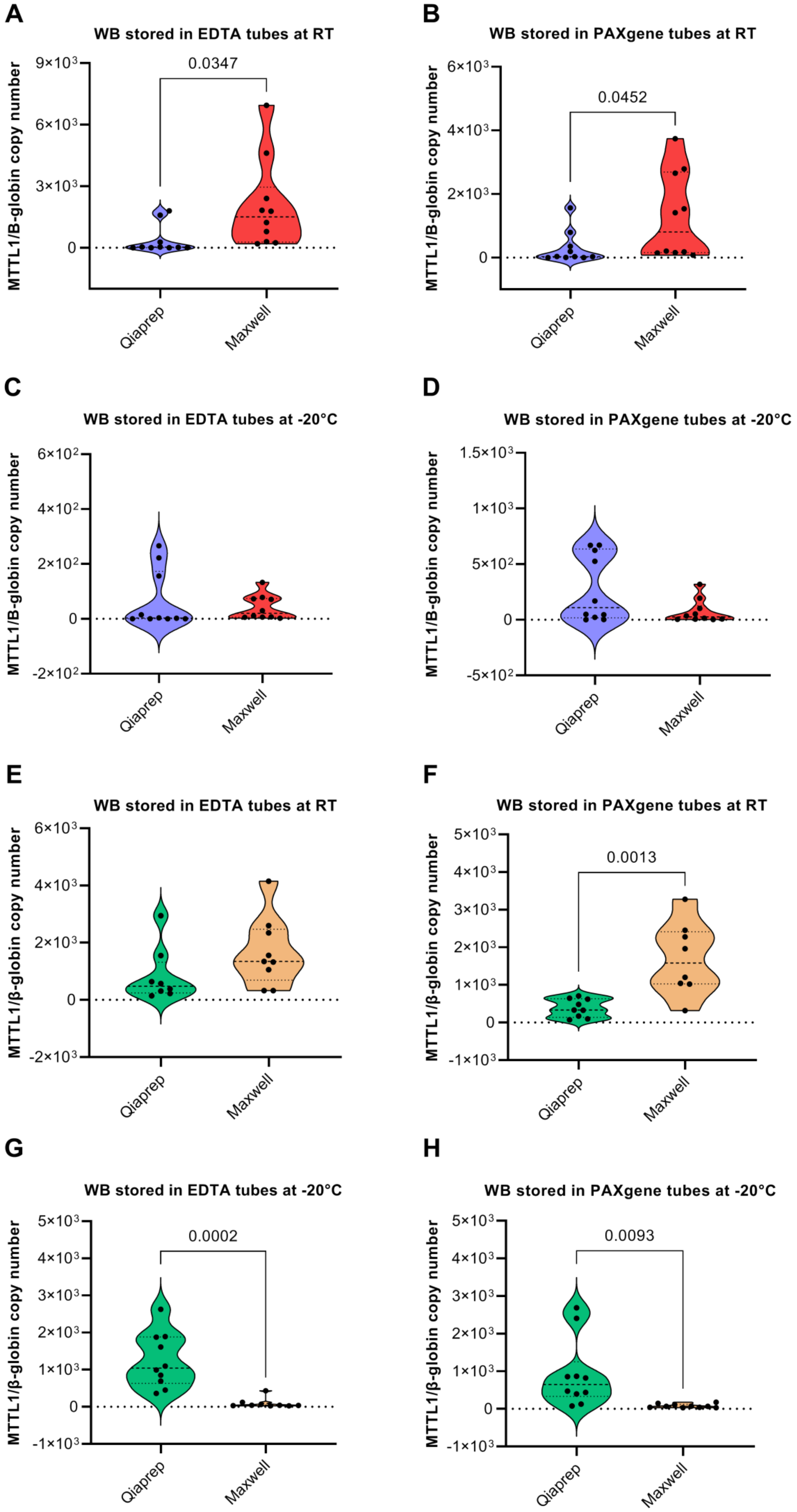
A
WB stored in EDTA tubes at RT
MTTL1/B-globin copy number
9×10³
6×10³
3×10³
0
0.0347
Qiaprep
Maxwell
B
WB stored in PAXgene tubes at RT
MTTL1/B-globin copy number
6×10³
4×10³
2×10³
0
0.0452
Qiaprep
Maxwell
C
WB stored in EDTA tubes at -20°C
MTTL1/B-globin copy number
6×10²
4×10²
2×10²
0
-2×10²
Qiaprep
Maxwell
D
WB stored in PAXgene tubes at -20°C
MTTL1/B-globin copy number
1.5×10³
1×10³
5×10²
0
-5×10²
Qiaprep
Maxwell
E
WB stored in EDTA tubes at RT
MTTL1/β-globin copy number
6×10³
4×10³
2×10³
0
-2×10³
Qiaprep
Maxwell
F
WB stored in PAXgene tubes at RT
MTTL1/β-globin copy number
5×10³
4×10³
3×10³
2×10³
1×10³
0
-1×10³
0.0013
Qiaprep
Maxwell
G
WB stored in EDTA tubes at -20°C
MTTL1/β-globin copy number
5×10³
4×10³
3×10³
2×10³
1×10³
0
-1×10³
0.0002
Qiaprep
Maxwell
H
WB stored in PAXgene tubes at -20°C
MTTL1/β-globin copy number
5×10³
4×10³
3×10³
2×10³
1×10³
0
-1×10³
0.0093
Qiaprep
Maxwell

Supplementary Figure S2. Comparison of methods for the isolation of mtDNA from whole blood and plasma. Comparison of mtDNA copy number present in whole blood samples of healthy subjects isolated with the Maxwell and Qiagen kit stored in (**A**) EDTA tubes at RT for 3h prior to processing, (**B**) PAXgene tubes at RT for 3h prior to processing, (**C**) EDTA tubes at -20 °C prior to processing, (**D**) PAXgene tubes at -20 °C prior to processing. Comparison of mtDNA copy number present in plasma samples of healthy subjects isolated with the Maxwell and Qiagen kit stored in (**E**) EDTA tubes at -20 °C prior to processing, (**F**) PAXgene tubes at -20 °C prior to processing. Comparison of mtDNA copy number present in whole blood samples of pancreatic cancer patients isolated with the Maxwell and Qiaprep kits stored in (**G**) EDTA tubes at RT for 3h prior to processing, (**H**) PAXgene tubes at RT for 3h prior to processing, (**I**) EDTA tubes at -20 °C prior to processing, (**J**) PAXgene tubes at -20 °C prior to processing. Comparison of mtDNA copy number present in plasma samples of pancreatic cancer patients isolated with the Maxwell and Qiagen kit stored in (**K**) EDTA tubes at -20 °C prior to processing, (**L**) PAXgene tubes at -20 °C prior to processing. Ten subjects were recruited in each cohort. Significant outliers were identified and omitted using Grubb's test (Alpha = 0.05). P-values < 0.05 indicate a statistically significant difference. Kit comparisons in terms of the yield of nuclear DNA in whole blood (as measured by a β-globin qPCR assay) is shown in Supplementary Figure S1, while the ratio of MTTL1 to β-globin recovered by the different kits in the different conditions is shown in Supplementary Figure S2.
