# Supplementary material for: Preanalytical Variables in the Analysis of Mitochondrial DNA in Whole Blood and Plasma from Pancreatic Cancer Patients
Source: Diagnostics (Basel). 2022 Aug 6;12(8):1905. doi: 10.3390/diagnostics12081905 (PMC9406772; doi:10.3390/diagnostics12081905)
Supplement: Supplementary file 1 [file diagnostics-12-01905-s001.zip › Supplementary Table S1.pdf]

Table S1. Mitochondrial DNA copy number (CN) in WB stored at different temperatures prior to processing.

Cohort	Tube	Subject	Room temperature		-20°C		Fold change in CN (-20°C/RT)
			MTTL1 CN	Stdev	MTTL1 CN	Stdev	
Healthy subjects	EDTA	1	4,67E+03	3,68E+02	4,90E+05	1,85E+05	104,92
		2*	6,43E+04	7,21E+03	3,10E+06	3,08E+06	48,33
		3	3,35E+04	2,27E+04	5,81E+05	2,49E+05	17,33
		4	8,66E+04	4,47E+04	8,03E+05	8,66E+04	9,27
		5	9,68E+04	4,07E+04	6,58E+05	2,47E+05	6,80
		6	1,03E+04	4,53E+03	5,80E+05	1,61E+05	56,50
		7	5,10E+03	1,73E+03	4,01E+05	9,45E+04	78,48
		8	3,66E+03	1,97E+03	2,28E+05	2,44E+05	62,22
		9	2,27E+05	2,13E+05	1,22E+06	4,72E+05	5,36
		10	1,21E+05	1,08E+04	1,35E+06	2,95E+05	11,13
	PAXgene	1	4,77E+05	1,29E+05	8,08E+05	4,05E+05	1,69
		2*	1,04E+07	5,01E+05	3,50E+06	9,88E+05	0,34
		3	8,14E+04	9,00E+03	1,23E+06	7,88E+05	15,13
		4	4,80E+05	4,09E+05	4,67E+05	1,53E+04	0,97
		5	3,66E+05	7,94E+04	6,47E+05	3,49E+05	1,77
		6	6,50E+04	3,41E+04	5,09E+05	2,53E+04	7,84
		7	6,78E+04	3,86E+04	3,66E+05	1,36E+05	5,40
		8	1,57E+04	1,35E+04	2,87E+05	7,14E+03	18,27
		9	4,21E+05	1,67E+05	1,15E+06	2,39E+05	2,73
		10	5,56E+05	2,20E+04	1,79E+06	2,18E+05	3,22
Cancer patients	EDTA	1	3,10E+05	7,03E+04	5,79E+05	1,87E+04	1,87
		2	1,24E+06	5,67E+05	1,49E+06	1,30E+05	1,20
		3	1,57E+06	7,26E+05	1,39E+06	2,71E+04	0,88
		4	1,05E+06	3,50E+05	8,05E+05	1,91E+05	0,77
		5*	1,49E+06	2,98E+05	3,66E+06	1,26E+05	2,45
		6	4,06E+05	5,19E+04	6,72E+05	1,16E+05	1,66
		7	6,31E+05	2,09E+05	5,83E+05	1,56E+05	0,92
		8	7,85E+05	1,61E+05	1,21E+06	2,58E+05	1,54
		9	1,24E+05	1,04E+05	1,43E+06	1,63E+04	11,51
		10	3,24E+05	1,08E+05	7,39E+05	5,63E+04	2,28
	PAXgene	1	4,89E+05	2,24E+05	6,30E+05	5,07E+04	1,29
		2	6,67E+05	2,46E+05	1,34E+06	9,48E+04	2,00
		3	1,53E+06	4,90E+05	1,82E+06	1,32E+05	1,19
		4	6,09E+05	1,04E+05	5,92E+05	5,74E+04	0,97
		5*	2,20E+06	1,06E+06	2,42E+06	1,43E+05	1,10
		6	8,37E+05	3,35E+04	8,88E+05	1,82E+05	1,06
		7	3,19E+05	1,20E+05	6,53E+05	6,76E+04	2,05
		8	8,82E+05	2,57E+05	9,81E+05	1,29E+05	1,11
		9	9,42E+05	8,23E+05	1,39E+06	4,07E+04	1,47
		10	4,73E+05	2,97E+04	1,20E+06	2,24E+05	2,53

*Significant outliers, as determined by the Grubb's test.
